# A Gene Prognostic Index Associated With Epithelial-Mesenchymal Transition Predicting Biochemical Recurrence and Tumor Chemoresistance for Prostate Cancer

**DOI:** 10.3389/fonc.2021.805571

**Published:** 2022-01-12

**Authors:** Dechao Feng, Xu Shi, Qiao Xiong, Facai Zhang, Dengxiong Li, Lu Yang

**Affiliations:** Department of Urology, Institute of Urology, West China Hospital, Sichuan University, Chengdu, China

**Keywords:** epithelial-mesenchymal transition, prostate cancer, tumor immune microenvironment, biochemical recurrence, tumor chemoresistance, immune checkpoint

## Abstract

**Background:**

We aimed to establish a novel epithelial-mesenchymal transition (EMT)-related gene prognostic index (EMTGPI) associated with biochemical recurrence (BCR) and drug resistance for prostate cancer (PCa).

**Methods:**

We used Lasso and Cox regression analysis to establish the EMTGPI. All analyses were conducted with R version 3.6.3 and its suitable packages.

**Results:**

We established the EMTGPI based on SFRP4 and SPP1. Patients in high-risk group had 2.23 times of BCR risk than those in low-risk group (*p* = 0.003), as well as 2.36 times of metastasis risk (*p* = 0.053). In external validation, we detected similar diagnostic efficacy and prognostic value in terms of BCR free survival. For drug resistance, we observe moderately diagnostic accuracy of EMTGPI score (AUC: 0.804). We found that PDCD1LG2 (*p* = 0.04) and CD96 (*p* = 0.01) expressed higher in BCR patients compared with their counterpart. For TME analysis, we detected that CD8+ T cells and M1 macrophages expressed higher in BCR group. Moreover, stromal score (*p* = 0.003), immune score (*p* = 0.01), and estimate score (*p* = 0.003) were higher in BCR patients. We found that EMTGPI was significantly related to HAVCR2 (*r*: 0.34), CD96 (*r*: 0.26), CD47 (*r*: 0.22), KIR3DL1 (*r*: −0.21), KLRD1 (*r*: −0.21), and CD2 (*r*: 0.21). In addition, we observed that EMTGPI was significantly associated with M1 macrophages (*r*: 0.6), M2 macrophages (*r*: −0.33), monocytes (*r*: −0.18), neutrophils (*r*: −0.43), CD8+ T cells (*r*: 0.13), and dendritic cells (*r*: 0.37). PHA-793887 was the common drug sensitive to SPP1 and SFRP4, and PC3 and DU145 were the common PCa-related cell lines of SPP1, SFRP4, and PHA-793887.

**Conclusions:**

We concluded that the EMTGPI score based on SFRP4 and SPP1 could be used to predict BCR for PCa patients. We confirmed the impact of immune evasion on the BCR process of PCa.

## Introduction

Aging population has already been a social dilemma worldwide and accounts for almost 20.8% of the population by 2044 (1). Prostate cancer (PCa), one of the most common age-related diseases, ranks the second most frequent cancer and the fifth leading cause of cancer death in men in 2020 (2). Thus, improving the prognosis and quality of life of such patients is an increasingly important area in urology with population aging globally. PCa often suffers from drug resistance and progress to castration-resistant state despite using new potent antiandrogen drugs, like abiraterone and enzalutamide (3, 4). It is known that metastatic castration-resistant PCa (MCRPC) is the leading cause of cancer death with an estimated mortality rate of almost 28% for 5-year survival (5). However, there has been much division between urologists on the molecular mechanism of metastasis and tumor chemoresistance in PCa.

Epithelial-mesenchymal transition (EMT) is endowed with a migratory phenotype, allowing cuboidal epithelial cells into motile mesenchymal phenotypes and enhancing invasiveness and migration (6). EMT is characterized by cadherin switching, which is the downregulation of E-cadherin and other epithelial markers, and the upregulation of markers of mesenchymal markers, such as N-cadherin, vimentin, and Snail (7). Furthermore, many studies demonstrate that EMT involves in the invasion, metastasis, and cancer resistance of PCa through a variety of mechanisms (7–10). Thus, it deserves to develop gene biomarkers or signature associated with EMT to predict prognosis of PCa. Recently, some researchers have proposed several gene signatures to predict biochemical recurrence (BCR) of PCa, but most of these signatures enrolled more than 5 genes, limiting their clinical applications (11–15). Here, for the first time, we developed and validated an EMT-related gene prognostic index based on only two genes predicting BCR and drug resistance in patients undergoing radical prostatectomy or radiotherapy. Tumor immune microenvironment (TME) of PCa was also analyzed.

## Methods

### Data Preparation

Our study has been registered in the ISRCTN registry (No. ISRCTN11560295). We removed batch effects of GSE62872 (16), GSE79021 (17), GSE32571 (18), and GSE116918 (19) from the Gene Expression Omnibus (GEO) database (20) (**Supplementary Figure S1**). EMT-related genes were obtained from MSigDB (21). Weighted gene coexpression network analysis was used to find cancer-related genes defined by lrl ≥0.3 and *p* adj. <0.0001. Differential genes between tumor and normal tissues of GSE62872 (16), GSE79021 (17), and GSE32571 (18) were considered as llogFCl ≥0.4 and *p* adj. <0.05. Subsequently, we obtained candidate genes through intersection of the above gene sets and determine the independently prognostic genes after LASSO and Cox regression analysis. We constructed the following formula: EMT-related gene prognostic index (EMTGPI) risk score = 0.2365 * SFRP4 + 0.2595 * SPP1. BCR-free survival was the primary outcome. We externally validated the prognostic value of EMTGPI using the TCGA database and GSE46602 (22). Furthermore, we examined the diagnostic efficacy of EMTGPI for tumor chemoresistance using GSE42913 (23).

### Function Analysis and m6A Analysis

We used the candidate genes to explore possible functions and signal pathways through gene ontology (GO) and Kyoto Encyclopedia of Genes and Genome (KEGG) analyses. GO analysis consisted of biological process, cell composition, and molecular function. Also, we classified the tumor patients in GSE116918 (19) into high- and low-risk groups based on the median of EMTGPI score, and gene set enrichment analysis (GSEA) was then conducted (24). We regarded *p*. adj. <0.05 and false-discovery rate ≤0.25 as statistical significance. The protein-protein interaction of SFRP4 and SPP1 was analyzed by GeneMANIA database (25). We conducted the m6A analysis as well.

### TME, Drug, and Cell Line Analysis

**Figure 1:**
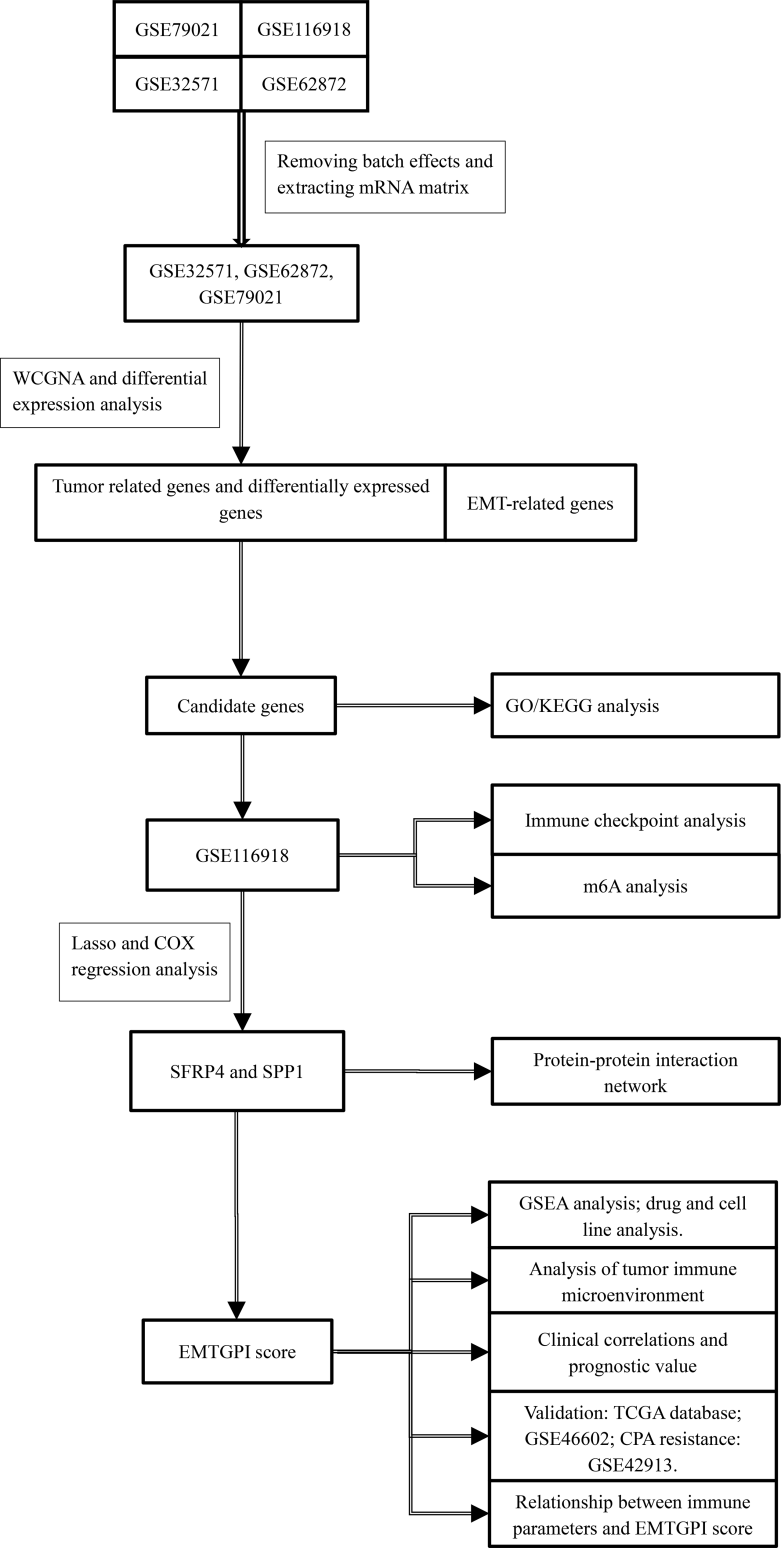
The study flowchart. WGCNA, weighted gene coexpression network analysis; GO, gene ontology; KEGG, Kyoto Encyclopedia of Genes and Genome; GSEA, gene set enrichment analysis; EMT, epithelial-mesenchymal transition; EMTGPI, EMT-related gene prognostic index; mRNA, message RNA.

We used the quanTIseq and ESTIMATE algorithms to analyze the immune infiltration levels of cells in TME (26–28). Immune checkpoint analysis was conducted as well. Differential expression between BCR and no-BCR group, prognosis of BCR-free survival, and Spearman’s analysis between parameters and EMTGPI were conducted for the above two analyses. We analyzed the potentially sensitive drugs of SFRP4 and SPP1 in the Cancer Therapeutics Response Portal (CTRP) and genomics of drug sensitivity in cancer (GDSC) through GSCALite (29). Moreover, the common cell lines of SFRP4, SPP1, and drugs were analyzed through the canSAR database (30). The study process can be seen in **Figure 1**.

### Statistical Analysis

We performed all analyses using software R 3.6.3 and its suitable packages. We utilized Wilcoxon test under the circumstance of nonnormal data distribution. Variables could be entered into multivariate Cox regression analysis if *p*-value <0.1 in the univariable Cox regression analysis. Survival analysis was conducted through log-rank test and presented as Kaplan-Meier curve. Also, the Spearman analysis was used to assess the correlations among continuous variables if they did not meet the Shapiro-Wilk normality test. Statistical significance was set as two-sided *p <*0.05. Significant marks were as follows: ns, *p* ≥ 0.05; ^*^ *p* < 0.05; ^**^ *p* < 0.01; ^***^ *p* < 0.001.

## Results

### EMTGPI Score and Its Clinical Values

**Figure 2:**
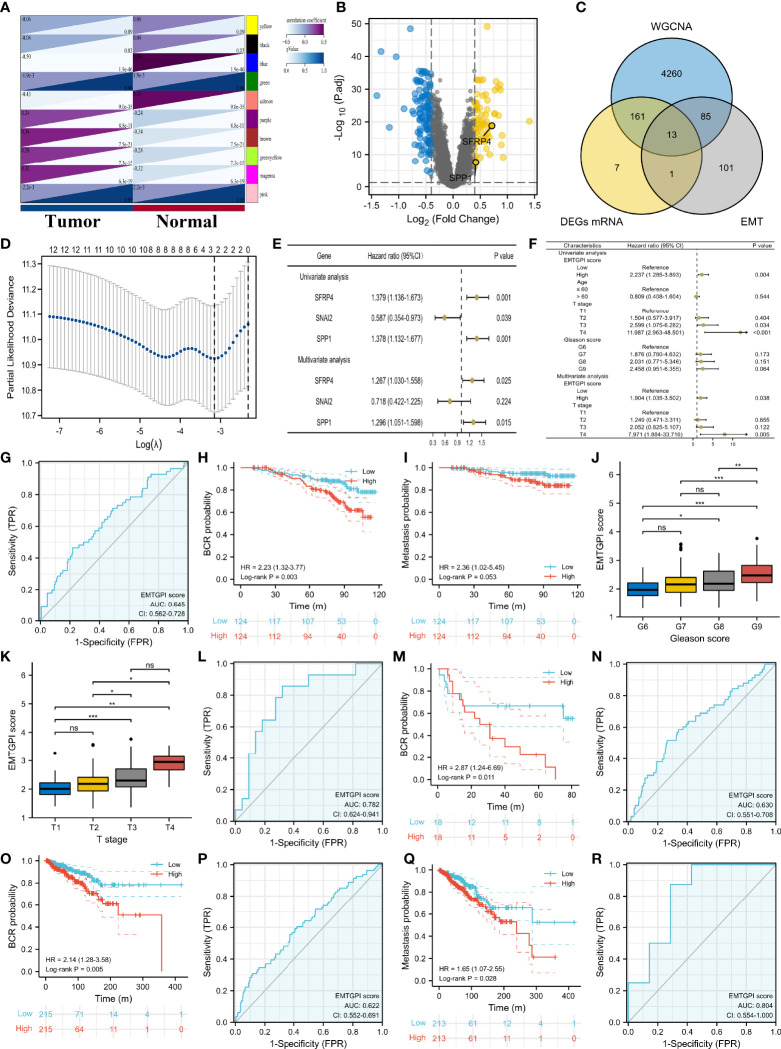
EMTGPI and its clinical values. **(A)** Modules and phenotype showing the modules associated with tumor and normal samples through WGCNA. **(B)** Volcano plot showing the DEGs. **(C)** Venn diagram showing the intersection of DEGs, tumor-related genes through WGNCA, and EMT-related genes. **(D)** Gene screening of LASSO regression. **(E)** Univariate and multivariate Cox analyses of genes after LASSO regression. **(F)** Univariate and multivariate Cox analyses of EMTGPI score and clinical parameters for BCR-free survival. **(G)** ROC curve of EMTGPI discriminating BCR from no BCR. **(H)** Kaplan-Meier curve for BCR free survival in terms of EMTGPI score (red lines = high risk; blue lines = low risk). **(I)** Kaplan-Meier curve for metastasis-free survival in terms of EMTGPI score (red lines = high risk; blue lines = low risk). **(J)** comparison between Gleason score groups for EMTGPI score. **(K)** Comparison between T-stage groups for EMTGPI score. **(L)** ROC curve of EMTGPI discriminating BCR from no BCR in GSE46602 (22). **(M)** Kaplan-Meier curve for BCR-free survival using GSE46602 (22) in terms of EMTGPI score (red lines = high risk; blue lines = low risk). **(N)** ROC curve of EMTGPI discriminating BCR from no BCR in TCGA database; **(O)** Kaplan-Meier curve for BCR-free survival using the TCGA database in terms of EMTGPI score (red lines = high risk; blue lines = low risk); **(P)** ROC curve of EMTGPI discriminating metastasis from no metastasis in TCGA database. **(Q)** Kaplan-Meier curve for metastasis-free survival using the TCGA database in terms of EMTGPI score (red lines = high risk; blue lines = low risk). **(R)** ROC curve of EMTGPI for drug chemoresistance. EMTGPI, epithelial-mesenchymal transition-related gene prognostic index; ROC, receiver operating characteristic; BCR, biochemical recurrence; WGCNA, weighted gene coexpression network analysis; EMT, epithelial-mesenchymal transition; mRNA, message RNA; DEGs, differentially expressed genes. *p 0.05; **p 0.01; ***p 0.001; ns, no significance.

We identified 13 candidate genes, and SFRP4 and SPP1 were used to construct the EMTGPI after LASSO and Cox regression analyses (**Figures 2A**–**E**). According to the median of EMTGPI score based on SFRP4 and SPP1, we divided the 248 patients undergoing radical radiotherapy in GSE116918 (19) into high- and low-risk groups. We observed that EMTGPI could be used as an independent factor of BCR-free survival after multivariate Cox regression analysis (HR: 1.904 (95% CI: 1.035–3.502), *p* = 0.038; **Figure 2F**). EMTGPI have lower diagnostic ability for distinguishing BCR from no BCR (AUC: 0.645; **Figure 2G**). Patients in high-risk group had 2.23 times of BCR risk than those in the low-risk group (*p* = 0.003; **Figure 2H**), as well as 2.36 times of metastasis risk (*p* = 0.053; **Figure 2I**). In addition, we observed that EMTGPI score increased with the increase of Gleason score (**Figure 2J**) and T stage (**Figure 2K**). In the GSE46602 (22) and TCGA databases, we detected similar diagnostic efficacy and prognostic value in terms of BCR-free survival (**Figures 2L**–**O**). Moreover, we found that high-risk patients had significantly higher risk of metastasis than their counterpart in the TCGA database (HR: 1.65 (95% CI: 1.07–2.55); **Figure 2Q**). For drug resistance, we observe moderate diagnostic accuracy of EMTGPI score (AUC: 0.804; **Figure 2R**).

### Function, Drug, and Cell Line Analysis

**Figure 3:**
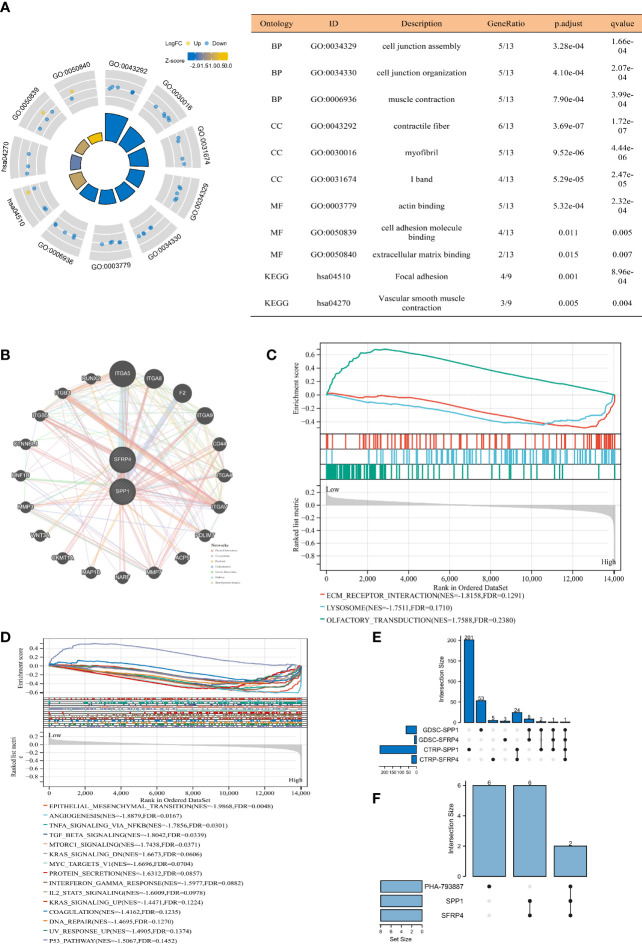
Function, drug, and cell line analysis. **(A)** GO and KEGG analyses. **(B)** Gene interaction network through GeneMANIA database (25). **(C)** GSEA C2 analysis. **(D)** GSEA hallmark analysis. **(E)** Upset plot showing common sensitive drug of SFRP4 and SPP1. **(F)** Upset plot showing common cell lines of SFRP4, SPP1, and PHA-793887. GO, Gene Ontology; KEGG, Kyoto Encyclopedia of Genes and Genome; GSEA, gene set enrichment analysis; GDSC, genomics of drug sensitivity in cancer; CTRP, the cancer therapeutics response portal; BP, biological process; CC, cell composition; MF, molecular function.

We found that the candidate genes primarily participated in cell junction assembly and organization, contractile fiber, actin binding, cell adhesion molecule binding, extracellular matrix (ECM) binding, focal adhesion, and vascular smooth muscle contraction (**Figure 3A**). The possible genes interacting with SFRP4 and SPP1 included ITGA5, ITGA8, RUNX2, NARFA, MMP7, CD44, and so on (**Figure 3B**). We observed that high-risk patients were highly enriched in ECM receptor interaction and lysosome (**Figure 3C**). The most possible hallmarks associated with high-risk patients were EMT, angiogenesis, TNFA signaling *via* NFKB, TGF beta signaling, motorc1 signaling, MYC targets V1, protein secretion, interferon gamma response, IL2 STATA5 signaling, DNA repair, p53 pathway, coagulation, ultraviolet (UV) response UP (genes upregulated in response to UV radiation), and so on (**Figure 3D**). PHA-793887 was the common drug sensitive to SPP1 and SFRP4 (**Figure 3E**), and PC3 and DU145 were the common PCa-related cell lines of SPP1, SFRP4, and PHA-793887 (**Figure 3F**).

### TME and m6A Analyses

**Figure 4:**
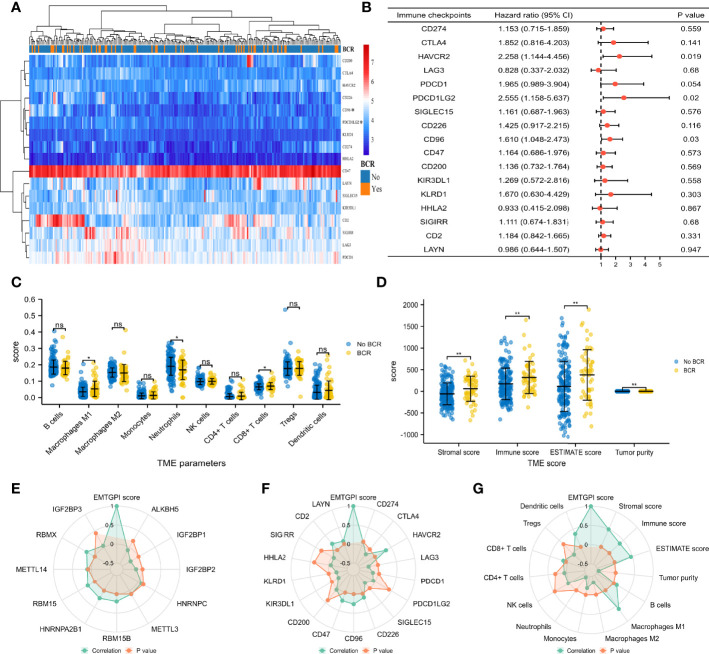
TME and m6A analysis. **(A)** Heatmap comparing immune checkpoints between BCR and no BCR group. **(B)** Univariate Cox analysis of immune checkpoints for BCR-free survival. **(C)** Comparison between BCR and no-BCR group for TME cells. **(D)** Comparison between BCR and no-BCR group for TME score. **(E)** Radar plot showing correlation between m6A-related genes and EMTGPI score. **(F)** Radar plot showing correlation between immune checkpoints and EMTGPI score. **(G)** Radar plot showing correlation between TME parameters and EMTGPI score. TME, tumor immune microenvironment; epithelial-mesenchymal transition-related gene prognostic index; BCR, biochemical recurrence. ns, no significance; *p < 0.05; **p < 0.01.

We found that PDCD1LG2 (*p* = 0.04) and CD96 (*p* = 0.01) expressed higher in BCR patients compared with their counterpart (**Figure 4A**), and both checkpoints were significantly associated with BCR-free survival (HRs were 2.555 and 1.610 for PDCD1LG2 and CD96, respectively; **Figure 4B**). For TME analysis, we detected that CD8+ T cells (*p* = 0.042) and M1 macrophages (*p* = 0.024) expressed higher in the BCR group, while neutrophils (*p* = 0.048) presented the opposite expression (**Figure 4C**). Moreover, stromal score (*p* = 0.003), immune score (*p* = 0.01), and estimate score (*p* = 0.003) were higher in BCR patients, while tumor purity (*p* = 0.003) was lower in BCR patients (**Figure 4D**). For m6A analysis, radar plot showed that EMTGPI was significantly associated with IGF2BP1 (*r*: −0.24), IGF2BP2 (*r*: −0.15), RBM15B (*r*: 0.2), HNRNPA2B1 (*r*: 0.23), RBM15 (*r*: 0.21), and RBMX (*r*: 0.25) (**Figure 4E**). We found that EMTGPI was significantly related to CTLA4 (*r*: −0.15), HAVCR2 (*r*: 0.34), LAG3 (*r*: −0.25), PDCD1 (*r*: −0.14), SIGLEC15 (*r*: −0.19), CD226 (*r*: 0.14), CD96 (*r*: 0.26), CD47 (*r*: 0.22), KIR3DL1 (*r*: −0.21), KLRD1 (*r*: −0.21), CD2 (*r*: 0.21), and LAYN (*r*: 0.12) (**Figure 4F**). In addition, we observed that EMTGPI was significantly associated with M1 macrophages (*r*: 0.6), M2 macrophages (*r*: −0.33), monocytes (*r*: −0.18), neutrophils (*r*: −0.43), CD8+ T cells (*r*: 0.13), and dendritic cells (*r*: 0.37) (**Figure 4G**).

## Discussion

With an aging population worldwide today, the problem of PCa receives increasing attention. The cadherin-switched property of EMT has been observed in more aggressive tumors, resulting in the decrease of intercellular adhesion, loss of epithelial cell polarity, de-differentiation into an amorphous cell, and increased motility (6, 31, 32). Downregulated E-cadherin and upregulated N-cadherin have been reported to be closely associated with progression and poor prognosis in PCa patients (33–35). In addition, EMT could promote the presence of CRPC and targeting N-cadherin inverts this process effectively (36). Due to these definitive evidences, the EMT-related biomarkers are warranted to be studied. In this study, we firstly developed and confirmed that EMTGPI could predict BCR probability and drug resistance effectively for PCa patients undergoing radical prostatectomy or radiotherapy. SFRP4 controls WNT signaling and is thought to play a role for tumor aggressiveness (37). SFRP4 overexpression in both androgen-dependent and androgen-independent cell lines resulted in a morphologic change to a more epithelioid cell type with increased localization of β-catenin and cadherins (E-cadherin in LNCaP, N-cadherin in PC3) to the cell membrane (38). Previous studies showed that SFRP4 overexpression was linked to advanced tumor stage, high classical/quantitative Gleason grade (*p* < 0.0001 each), lymph node metastasis (*p* = 0.0002), and a positive surgical margin (*p* = 0.0017), and SFRP4 expression was an independent predictor of recurrence after prostatectomy (HR = 1.35; *p* = 0.009) (22, 37). In addition, SPP1 is a cardinal mediator of tumor-associated inflammation and facilitates metastasis. Pang et al. (39, 40) showed that SPP1 could promote enzalutamide resistance and EMT activation in castration-resistant prostate cancer *via* PI3K/AKT and ERK1/2 pathways. Compared with the previous studies (11–15), we included two different genes in our study, and these two genes have confirmed their roles in the progression of PCa and are closely associated with the EMT. Moreover, we provided a simpler prognostic gene formula from the perspective of EMT.

We also indirectly demonstrated that EMT is implied in the process of PCa progression and chemoresistance, which was similar to the previous study, indicating that EMT drives docetaxel resistance and promotes the risk of recurrence in PCa patients (41).

Previous studies indicated that the plasticity of EMT tumor cells, which are in a transitory state between epithelial and mesenchymal programs, made it possible to accomplish the invasion-metastasis cascade, rather than the mesenchymal-like tumor cells that have fully completed the EMT program (32, 42). Compelling evidence showed that transforming growth factor β (TGF-β) is a potent inducer of EMT and chemoresistance in multiple cancers, including PCa, through small mothers against decapentaplegic homolog-independent or homolog-dependent signaling pathway (9, 32, 43–47). We further confirmed the impact of EMT and TGF-β signaling on PCa through ECM interaction according to the GSEA analysis.

The antitumor immune cells include CD8+ cytotoxic T cells and effector CD4+ T cells, natural killer cells, dendritic cells, M1 macrophages, and N1 neutrophils in the TME (48). Dendritic cells secreted chemokines like CXCL9 and CLCL10, and CD8+ cytotoxic T cells were recruited into the inflammatory niches through the expression of CXCR3 (48–50). M1 macrophages within TME are usually considered protective cells due to their proinflammatory function and tumor cell killing (48). Also, we found EMTGPI was highly associated with HAVCR2, CD96, CD47, and CD2. All of the above immune checkpoints could contribute to immune evasion through inhibiting the function of host antitumor cells, such as T cells and natural killer cells (51–54). Given the higher score of M1 macrophages, CD8+ T cells, immunity, and estimate in the BCR group, we proposed the presence of immune evasion in the progression of PCa. In most solid tumor, abundant matrix is usually associate with poo prognosis, and the prostate stroma accounts for more proportion in the process of prostate growth and differentiation (55, 56), which has been called “reactive stroma” and are used to assess PCa-specific mortality in diagnostic prostate needle biopsies (57). In this study, we also observed that stromal score was higher in the BCR patients than no-BCR patients, and this score was positively associated with the EMTGPI. In terms of tumor purity, previous studies showed that low tumor purity was associated with unfavorable prognosis and immune-evasion phenotype in gastric cancer (58), and most recognized prognostic indicators were no longer significantly effective under different tumor purity conditions (59). Similarly, we observed that tumor purity was lower in the BCR patients than no-BCR patients, and this score was negatively associated with the EMTGPI, which both suggested that the tumor purity might play an important role in PCa treatment and prognosis assessment.

Previous study showed that HNRNPA2B1, an m6A methylation regulator, was not only highly expressed in patients with high Gleason score but also significantly associated with unfavorable BCR-free survival in PCa patients (60). Similarly, we observed a significantly positive correlation between HNRNPA2B1 and EMTGPI. PHA-793887 could inhibit the proliferation of multiple tumor cell lines (such as PC3 and DU145) *in vitro* and *in vivo* through inducing arrest of cell cycle and inhibiting phosphorylation of Rb and nuclear phosphoprotein (61, 62). In this study, we found that PHA-793887 might be a potentially sensitive drug, and PC3 and DU145 could be the studied cell lines for PCa patients. This study had several limitations. Firstly, the prognostic value of EMTGPI score was different between the GSE116918 (19) and TCGA databases for metastasis-free survival. We thought the different treatments used contributed to the result. Secondly, the estimated drug and cell lines were needed to be further confirmed *in vitro* and *in vivo*. Furthermore, the diagnostic effect of EMTGPI for tumor chemoresistance was warranted to be validated in larger samples as well.

## Conclusions

We concluded that the EMTGPI score based on SFRP4 and SPP1 could be used to predict BCR for PCa patients. We confirmed the impact of immune evasion on the BCR process of PCa.

## Data Availability Statement

The original contributions presented in the study are included in the article/**Supplementary Material**. Further inquiries can be directed to the corresponding author.

## Author Contributions

Concept: DF. Data collection and processing: DF, XS, and QX. Software: DF, FZ, and DL. Supervision: LY. Manuscript draft: DF. Manuscript editing and review: all authors. All authors contributed to the article and approved the submitted version.

## Funding

This program was supported by the National Natural Science Foundation of China (Grant Nos. 81974099, 82170785, 81974098, and 82170784), programs from Science and Technology Department of Sichuan Province (Grant No. 21GJHZ0246), Young Investigator Award of Sichuan University 2017 (Grant No. 2017SCU04A17), Technology Innovation Research and Development Project of Chengdu Science and Technology Bureau (2019-YF05-00296-SN), Sichuan University–Panzhihua science and technology cooperation special fund (2020CDPZH-4). The funders had no role in the study design, data collection or analysis, preparation of the manuscript, or the decision to publish.

## Conflict of Interest

The authors declare that the research was conducted in the absence of any commercial or financial relationships that could be construed as a potential conflict of interest.

## Publisher’s Note

All claims expressed in this article are solely those of the authors and do not necessarily represent those of their affiliated organizations, or those of the publisher, the editors and the reviewers. Any product that may be evaluated in this article, or claim that may be made by its manufacturer, is not guaranteed or endorsed by the publisher.

## Supplementary Material

Supplementary Figure 1 The process of merging four GEO datasets. **(A)** Upset plot; **(B)** box plot before removing batch effects; **(C)** box plot after removing batch effects; **(D)** density plot before removing batch effects; **(E)** density plot after removing batch effects; **(F)** umap plot before removing batch effects; **(G)** umap plot after removing batch effects.
