# Supplementary material for: A Gene Prognostic Index Associated With Epithelial-Mesenchymal Transition Predicting Biochemical Recurrence and Tumor Chemoresistance for Prostate Cancer
Source: Front Oncol. 2022 Jan 12;11:805571. doi: 10.3389/fonc.2021.805571 (PMC8790245; doi:10.3389/fonc.2021.805571)
Supplement: Supplementary Figure 1 — The process of merging four GEO datasets. (A) Upset plot; (B) box plot before removing batch effects; (C) box plot after removing batch effects; (D) density plot before removing batch effects; (E) density plot after removing batch effects; (F) umap plot before removing batch effects; (G) umap plot after removing batch effects. [file DataSheet_1.docx]

Supplementary figure 1. The combined process of the four GEO datasets.
